# Massage perceptions and attitudes of undergraduate pre-professional health sciences students: a cross-sectional survey in one U.S. university

**DOI:** 10.1186/s12906-020-03002-6

**Published:** 2020-07-08

**Authors:** Niki Munk, Abby Church, Donya Nemati, Samantha Zabel, Amber R. Comer

**Affiliations:** 1 Department of Health Sciences, School of Health and Human Sciences, Indiana University – IUPUI, 1050 Wishard Blvd, Indianapolis, IN 46202 USA; 2 Australian Research Centre in Complementary and Integrative Medicine (ARCCIM), Massage & Myotherapy Australia Fellow and Visiting Faculty of Health, University of Technology Sydney, Sydney, Australia; 3 Indiana University Robert H. McKinney School of Law, 530 W. New York St, Indianapolis, IN 46202 USA; 4 Regenstrief Institute, Inc., 1101 W. 10th St, Indianapolis, IN 46202 USA

**Keywords:** ATOM scale, CAM, CIM, Gender bias, Massage therapy, Professional touch, Referral

## Abstract

**Background:**

Attitudes and beliefs about massage therapy have been explored among health professionals and health profession students, but not for undergraduate preprofessional health sciences students.

**Methods:**

This cross-sectional survey sought to determine pre-professional health students’ attitudes and perceptions toward massage therapy and determine the extent demographic variables such as age, gender, race, along with lifetime massage experience are associated with neutral/negative perceptions.

**Results:**

*N* = 129 undergraduate students completed the Attitudes Toward Massage scale and 7 supplemental items pertaining to sexuality and therapist gender preference along with questions regarding lifetime massage utilization. Prevalence of massage therapy utilization was 35.6% (lifetime) and 18.6% (last 12-months). Overall, positive attitudes towards massage therapy was observed with participants reporting massage experience expressing more positive massage attitudes (lifetime; *p* = 0.0081, the past 12 months; *p* = 0.0311). Participants with no massage experience were more likely to report neutral/negative attitudes toward massage (*p* = 0.04). Men were more likely to prefer their massage therapist to be of the opposite sex (38.9%) compared to women (2.1%) (*p* = < 0.0001). Men were less confident than women in their concern of becoming sexually aroused during massage (*p* = 0.0001) and in the belief that massage is sexually arousing (*p* = 0.048). Both genders expressed comfort with female and/or male massage therapists, but if given a choice, both prefer a female massage therapist.

**Conclusions:**

Undergraduate pre-professional health sciences students have generally positive attitudes towards massage therapy however more research is needed regarding implicit gender bias and/or preferences. This work should inform future research designs examining the impact of attitudes and beliefs on patient referrals to massage therapy.

## Background

Complementary and alternative medicine (CAM) involves health related practices such as massage therapy, acupuncture, chiropractic therapy, meditation, and yoga that are not considered a part of conventional medical treatments [1, 2]. Although CAM is not conventional medicine, various practices are used broadly by patients [3, 4] and referrals to CAM from healthcare professionals has increased [5]. A newly published analysis of a broad and nationally represented survey indicates as many as 53% of U.S. office-based physicians made a least one patient referral to a CAM practice within the past year [6]. One of the most popular CAM practices is massage therapy [3, 7, 8]. A national survey found that 12.8% of U.S. adults utilized therapeutic massage in their lifetime and approximately 15.4 million (7%) of adults had used massage therapy for disease prevention, musculoskeletal pain, and wellness in the past 12 months [3].

Crawford and colleagues [8] describe massage therapy as involving “the systematic manipulation of soft tissue with the hands that positively affects and promotes healing, reduces stress, enhances muscle relaxation, improves local circulation, and creates a sense of well-being” (p. 1355). The most common reasons people seek massage therapy are musculoskeletal pain, disease prevention, and wellness [3]. Massage therapy has been found to alleviate pain, reduce the burden of chronic pain and pain medication use, improve anxiety and sleep, support emotional wellbeing, and contribute to stress relief [9–12]. Although massage therapy has been shown to have many positive benefits, its advantages are not widely understood or accepted by the medical community [13] and the benefits of massage therapy are not taught in-depth during conventional allopathic medical education [14–17]. Because knowledge about, experience with, and understanding of practices impacts care providers’ patient referrals, health professionals’ attitudes and beliefs about CAM generally and massage therapy specifically is important [18–21].

The current attitudes and perceptions of CAM including massage therapy among health professionals is not inherently negative [22, 23] and studies have found that health professionals are interested in learning more about the practices in part, to better prepare them for referrals in practice [1, 18]. Although the attitudes and perceptions of massage therapy have been studied among graduate health practitioner populations [2, 24], there is a gap of knowledge about massage therapy attitudes and perceptions among undergraduate pre-professional health students. Undergraduate health sciences students are the future professionals within their respective fields and identifying their attitudes and perceptions toward emerging complementary practices such as massage therapy will allow for the development of education interventions to increase knowledge about the practice and benefits of massage therapy.

The current study is the first of its kind to address the knowledge gap about pre-professional health students and their attitudes and perceptions about massage therapy. Specifically, this study sought to determine pre-professional health students’ attitudes and perceptions toward massage therapy and determine the extent demographic variables such as age, gender, and race, along with lifetime experience of massage are associated with a neutral or negative perception of massage therapy.

## Methods

To examine undergraduate pre-professional health science students’ perceptions and attitudes regarding massage therapy, a cross-sectional survey was conducted using the Attitudes Toward Massage (ATOM) scale, a validated instrument [25]. The study was approved by the institution’s Institutional Review Board (IRB approval #1903143220). All data was deidentified and entered into and stored in REDCap (Research Electronic Data Capture), a secure, web-based application designed to support data capture for research studies, providing: an intuitive interface for data entry; audit trails for tracking data manipulation and export; and automated export procedures [26].

### Setting

The survey was conducted across eight face-to-face Health Science courses at a large urban university in the Midwest between April 1 and April 12, 2019. The seven lecturers and/or professors teaching face-to-face courses were contacted by the study team for permission to hand out the survey (Additional file 1), with six (86%) agreeing to allow access to their students. A research assistant went to each class on a designated day to administer the survey.

### Participants and procedures

Undergraduate pre-professional health science students enrolled in at least one face-to-face course, who were able to read and write in English were included in this study. Health Sciences majors were selected for this study as the majority of these students indicate that they intend to attend graduate school and become a health care professional. There were 526 total students enrolled in the undergraduate health sciences program, of which 180 were enrolled in a face-to-face class during the study period. Due to the adult nature of the supplemental questions relating to sexuality and gender, students were required to be over the age of 18. Paper surveys were given to all students present in the classroom. Those who did not wish to participate, or who had already participated in the study were required to return the blank survey paper to ensure that no individual could be identified as participating or declining. To ensure students did not participate in the survey more than one time, students were asked a screening question prior to participation and were excluded if they had previously taken the survey. Students were verbally consented prior to being given a survey; final consent was implied when surveys were submitted. Data was entered into a password-protected REDCap database and rechecked by at least two co-investigators for accuracy.

### Survey

The ATOM survey is a 9-question scale with several supplemental questions related to sexuality and gender. The ATOM has been validated to yield a reliable Global score of an individual’s overall attitude toward massage with a Cronbach α = .85 and has been used in several studies [25, 27–30]. Responses are scored using a 5-point Likert scale, resulting in survey scores ranging from 9 to 45. Higher ATOM scores indicated more positive attitudes toward massage but no prior studies have specified at what level a score is considered positive, neutral, or negative. For this study’s purposes, scores were categorized into positive, neutral, and negative by dividing the 36-point range into 5 parts, correlating to the five answer choices for each question. Scores below 22 were considered negative (majority disagree or strongly disagree), from 23 to 31 were neutral, and 32 and up were positive (majority agree or strongly agree). The question “Receiving a massage would make me nervous” (question 8) was reverse scored, with “disagree” or “strongly disagree” indicating a positive response.

### Data analysis

Demographic data of all participants was collected and summarized using descriptive statistics. The primary objective was to determine the proportion of undergraduate pre-professional health sciences students who have a neutral or negative perception of massage. Secondary objectives included determining if demographic variables such as gender, year in school, age, race, and history of massage, are associated with a neutral or negative perception of massage among undergraduate pre-professional health sciences students. Statistical tests included frequency, chi-square, and univariate ANOVA against demographic variables, ATOM score, and responses to the supplemental questions. A logistic regression was performed on responses to the supplemental questions. Chi-square analysis were conducted to determine attitudes and perceptions of students toward massage. Neutral and negative attitudes were assessed with variables of age, gender, race, class year, and massage experience. All analyses were performed using SAS 9.4 (SAS Institute, Cary, NC), and *p*-values less than 0.05 were considered statistically significant. The dataset supporting the conclusions of this article is available upon reasonable request from the corresponding author.

## Results

A total of 129 students completed the survey yielding a response rate of 72%. Table 1 contains participant demographic descriptors. The students ranged in age from 18 to 47 (mean age = 21.7 years, S.D. 4.2), with the majority falling within the traditional undergraduate college student age of 18–23 year’s old [31]. The gender make-up of the participant sample is primarily female (72%) and a majority identify as White (64%). Participants spanned the freshman – senior undergraduate continuum with the smallest proportion indicating sophomore status (14%) and a majority of participants (65%) indicating they were upper classman (either junior [31%] or senior [34%]). Slightly over one-third of participants indicated having had a professional massage experience in their lifetime (36%) while only 19% (*n* = 24) indicated having had a massage within the past year. Demographic differences existed between those with and without massage experience (data not shown). Specifically, higher proportions (60% vs. 31%) of students older than typical for undergraduate students (> 23) indicated having one or more professional massages in their lifetime (χ^2^ = 5.96; *p* = 0.01). Likewise, those indicating they were seniors had higher proportions of lifetime massage experience (52% vs. 30, 22, and 28%, respectively) compared to all other class delineations (χ^2^ = 8.298; *p* = 0.04).

**Table 1:** Demographic characteristics of participants (*N* = 129)

Variables	n (%)
Age	
*Mean Age Years*	21.7 (4.2)
*18–23 Years*	108 (84.4)
*> 23 Years*	20 (15.6)
Gender	
*Male*	36 (28.1)
*Female*	92 (71.9)
Race	
*White*	81 (64.3)
*Non-White*^a^	45 (35.7)
Ethnicity	
*Hispanic*	15 (11.7)
Class Year Delineation	
*Freshman*	27 (20.9)
*Sophomore*	18 (14.0)
*Junior*	40 (31.0)
*Senior*	44 (34.1)
Massage Experience	
*Ever in Lifetime (yes)*	46 (35.7)
*Past year (yes)*	24 (18.6)

^a^ Non-White categorization included those who indicated Black (14.1%), Asian (5.5%), Multi-racial (3.9%), Other (11.7%) and Missing (1.6%)

Additional file 2: Table S2 contains responses to each of the ATOM scale questions and supplement items. The majority of participants agreed that massage is a serious form of therapy (79%), good for the mind and body (92%), and good for promoting health and well-being (89%). The vast majority of respondents did not respond favorably to the prompt “I like to be touched by other people,” (82%), and slightly under two-thirds of respondents (61%) indicated massage should be covered by health insurance.

Predictors associated with high ATOM scores and neutral/negative massage attitudes are presented in Table 2. Respondents who reported massage experience within their lifetime (*p* < 0.00) and/or in the past 12 months (*p* = 0.03) had significantly higher ATOM scores indicating they have more positive attitudes towards massage compared to those without massage experience within their lifetimes or in the past year, respectively. White participants were more likely to have a higher ATOM score than other racial groups (*p* = 0.03). Only one variable was associated with neutral/negative massage attitudes in the sample. Specifically, higher proportions of neutral/negative massage attitudes were reported among those with no massage experience within their lifetimes compared to those with massage experience (*p* = 0.04).

**Table 2:** Demographic Predictors for ATOM Global Scores and Neutral/Negative Massage Attitudes and Perceptions Indication

Variables	ATOM Score Mean (SD) 36.1 (5.0)	***p***-value	Negative/Neutral n (%) *n* = 23 (18%)	***p***-value
Age				
*18–23 Years*	36.2 (5.1)	0.88	21 (91.3)	0.31
*> 23 Years*	36.0 (4.8)		2 (8.7)	
Gender				
*Male*	35.7 (5.1)	0.63	5 (21.7)	0.45
*Female*	36.2 (5.0)		18 (78.3)	
Race				
*White*	36.9 (4.4)	0.03*	12 (52.2)	0.18
*Non-White*	34.8 (5.8)		11 (47.8)	
Class Years				
*Freshman*	35.5 (4.2)		6 (26.9)	
*Sophomore*	35.7 (6.1)	0.38	4 (17.4)	0.57
*Junior*	35.4 (4.7)		8 (34.8)	
*Senior*	37.2 (5.2)		5 (21.7)	
Massage Experience				
*Lifetime Yes*	37.7 (4.8)	0.008*	4 (17.4)	0.04*
*Lifetime No*	35.2 (4.9)		19 (82.6)	
*Past year Yes*	38.1 (5.1)	0.03*	2 (8.7)	0.18
*Past year No*	35.6 (4.9)		21 (91.3)	

* Significant *p*-values at ≤0.05

Differences in supplemental ATOM item responses were examined based on lifetime massage experience (Additional file 3: Table S4) and gender (Additional file 4: Table S5). There was no statistical difference between those with and without massage experience regarding questions about massage being sexually arousing (*p* = 0.19) or comfort with male or female massage therapists (*p* = 0.50 and *p* = 0.95, respectively). However, there was a significant difference between groups when it came to preference for same-sex therapists (*p* = 0.03). Specifically, higher proportions of those without massage experience indicated neutral preference for same sex massage therapists (65% vs. 41%) whereas those with massage experience had higher proportions of respondents either agreeing (37% vs. 23%) or disagreeing (22% vs. 12%) with same sex preferences for their massage therapists.

Supplemental ATOM item comparisons between male and female participants yielded several significant differences (Additional file 4: Table S5). Men were significantly more likely to prefer massage therapists of the opposite sex compared to women (39% vs. 2%; *p* < 0.00). Conversely, only 6% of men strongly agreed to a preference for a same sex massage therapist compared to 37% of women (*p* < 0.00). No statistical differences were observed between genders regarding being ‘comfortable’ with male or female massage therapists. A larger proportion of women (92%) expressed disagreement with the concern that they might become sexually aroused during a massage compared to 61% of men (*p* < 0.00). However, males were significantly less likely than women to disagree with the statement that massage is sexually arousing (58% vs. 79%; *p* = 0.05).

## Discussion

This study is the first to examine the attitudes and perceptions of undergraduate pre-professional health focused students with regard to therapeutic massage. Our findings suggest that undergraduate pre-professional health sciences students’ attitudes and perceptions toward massage therapy are generally positive. Moreover, the usage of massage therapy in this population (35.6%) is consistent with previous studies reporting from 22.9 to 35% of lifetime massage experience among college students generally and medical students specifically [1, 7, 22, 23]. The overall positive attitudes toward massage in this study might be attributed to the general popularity of therapeutic massage that places it among one of the most popular practitioner based complementary practices in U. S population [3].

A general perception that massage therapy provides health benefit is supported by this study with a vast majority of participants (89%) indicating the belief that receiving regular massage would be good for promoting health and well-being along with the belief held among a majority of participants (79%) that massage is a serious form of therapy. Contrary to expectations, nearly 40% of the participants did not support the idea of covering massage therapy by health insurance. This finding may indicate pre-professional health focused students are hesitant to view massage therapy alongside other health and rehabilitation professions. Such a hesitancy may be reinforced by the low number of insurance providers that currently cover or reimburse massage services [32]. Nearly all policies that cover massage only permit coverage or reimbursement under certain circumstances, such as with a doctor’s prescription or for certain conditions, and 27% excluded massage entirely while allowing other CAM services such as chiropractic or acupuncture [32]. It is possible that more insurance companies will begin covering massage therapy more comprehensively; reflecting more complementary practice inclusive healthcare coverage policies such as those within the Veteran’s Health Administration and other large healthcare systems [33, 34]. As future workers within an evolving healthcare field, education for pre-professional health focused students should include exposure to the philosophy behind, practice landscape, and evidence base for massage therapy and other CAM practices to best serve patient needs as resources to available care.

Among the demographic data collected, massage experience and gender had the most impact on ATOM score responses. Participants reporting massage experience were more likely to have a positive attitude toward massage and had higher global ATOM scores than those without massage experience. The opposite was reflected for those who did not have prior massage experience in that they were more likely to have a neutral/negative opinion of massage. It has been reported that familiarity and experience with different CAM modalities is associated with the positive attitudes toward the practices [2]. Therefore, it is not surprising that we observed significant statistical differences between ATOM scores for those with and without massage experiences. What is still unknown and rather important, is the impact of professional health providers’ massage attitudes and beliefs on referral intention and practice for patients with conditions that may benefit from massage treatment. Positive CAM beliefs have not always predicted usage and referrals in health professions such as nursing [35] and research focused on yoga found personal experience with the practice was a factor in its consideration as a referral option among health professions students [21]. Although massage attitudes in this sample of pre-professional health sciences students is positive overall, it cannot and should not be assumed they will refer patients who would benefit to massage therapy services. Results from this study considered within the context of relevant referral pattern literature points to a need for future research to examine the relationship between massage attitudes and appropriate referrals as well as studies examining the extent to which neutral or negative massage attitudes held by those naïve to massage are impacted by professional massage experiences.

Some of this study’s most interesting findings relate to massage attitudes concerning sexuality and gender preference for massage therapists. A similar proportion of female and male participants were comfortable with both male and female massage therapists, however when given the choice, both genders expressed a preference for a female therapist at a significant level. These results confirm a previous pilot study conducted in general youths aged 14–23 years [36] and is consistent with other literature [25, 37] providing additional evidence to suggest there may be a gender bias against male massage therapists from a career standpoint. This bias against male therapists may be justifiable considering the intimate nature of massage [38] and the accepted social role of women as more caring and sensitive compared to men [25]. This could potentially have implications for the massage therapy workforce. Male therapists frequently struggle to be as accepted by clients as their female therapist counterparts because of their gender, as men are often viewed as more sexually aggressive than women [37]. Gender bias exists in healthcare settings but are most often detrimental to female students, providers, and patients [39]. Pre-professional health sciences students of both genders should be aware of the potential for gender biases and associated implications and have educational experiences reinforcing related awareness.

Two of the ATOM supplemental questions, “I’m afraid I might become sexually aroused during a massage,” and “Receiving massage is often sexually arousing,” were answered neutrally and negatively at a significant level in our sample in contrast to prior findings [25]. These findings’ differences may be due to the over 10 years difference between our work and Moyer and colleagues’ work [25] and differences in study populations. However, through critical reflection and dialogue among the study team, it was determined that these questions may have been asked or understood by the respondents in a way that reversed the intent of the question and potentially led the respondent to consider the massage therapist threatening or imposing. Future research may include re-validating the ATOM scale and reconsidering the wording of supplemental questions to be less vague. Specifically, qualitative research methodology seems particularly warranted for related future research to ensure key issues are identified with regard to how questions should be worded to get valid and reliable prevalence estimates for these issues.

Finally, while overall ATOM scores were positive, 81.4% of respondents noted a neutral or negative response to the question of liking to be touched by other people. While certainly different from comfort with or liking to touch other people, this is an interesting finding in the population of pre-professional health science students because many will be going into professions where they will need to or be required to touch others as an integral part of their future profession. Many of the allopathic health fields are considered “caring professions,” in which it is the job of the person to care for another individual; so touching is intrinsic to that profession. It would be difficult to conduct many of the professions in the allopathic health fields without physical touch. Touch is an instrumental but little understood component of healthcare including being used as a form of communication and as a means to invoke therapeutic change [40]. Professional touch within a healthcare setting has multiple meanings and influences [40] and pre-professional health sciences students should have educational opportunities to increase awareness for related boundaries and to prepare themselves for the necessities of touch and being touched which come with working in a healthcare environment.

### Limitations

Sound methodology was used for this study resulting in a reasonable sample size with high response rate in an appropriate population. Despite this study’s strengths, several limitations exist. One key limitation resided within the ATOM scale; ATOM score interpretations or categorizations do not exist in the literature. For this study’s purposes, scores were categorized in a rational and logical way to denote respondents as generally positive, neutral, or negative. In addition, there was little diversity within the sample and respondents were mostly white and female although these distributions are reflective of pre-health care professional students majoring in health sciences. It is not known what the impact of additional diversity would or would not have done for this study’s results. Finally, this was one cross-sectional survey conducted at one Midwestern university in the United States. Findings from cross-sectional research cannot determine causation and it is somewhat impossible to generalize this study to other universities that do not fit the demographic and geographic make-up of this study setting. Replication studies at other universities with various demographic representation are needed to determine if these results are typical.

## Conclusions

Massage therapy has many benefits and is one of the most utilized and referred to of the various CAM practices. Clinician attitudes and beliefs about massage therapy may contribute to their willingness to refer patients who would benefit from such practices. Attitudes and beliefs about massage therapy have been explored among health professionals and health profession students, but not for undergraduate pre-professional health sciences students. This cross-sectional survey sought to determine pre-professional health students’ attitudes and perceptions toward massage therapy and determine the extent demographic variables such as age, gender, race, along with lifetime massage experience are associated with neutral/negative perceptions. Approximately 36% of participants had some professional massage experience within their lifetime and just under 20% reported having had a professional massage in the past 12 months. Massage attitudes were generally positive among the sample but participants with massage experience within their lifetimes had higher ATOM scale scores indicating more positive massage attitudes compared to those without massage experience. Both genders expressed comfort with female and male massage therapists, but when given a choice, both genders indicated preference for a female massage therapist. As future healthcare professionals, pre-professional health focused students in programs such as health sciences should be educated in the practice landscape and evidence base for prevalent CAM practices such as massage therapy. Pre-professional healthcare students will be relied upon as partners in health by their patients and will serve as a valuable resource for referrals, recommendations, and healthcare navigation. Research is needed to understand the impact of attitudes and beliefs on referral intention and practices among all levels of healthcare provider students and professionals with regard to massage therapy and the extent to which massage experience impacts attitudes and beliefs.

## Supplementary information

**Additional file 1.**

**Additional file 2.**

**Additional file 3.**

**Additional file 4.**

## Data Availability

The PDF of the data collection instrument is available as supplementary information to this manuscript (Additional file 1). The dataset supporting the conclusions of this article are available from the corresponding author on reasonable request.

## Abbreviations

ANOVA: analysis of variance
ATOM: Attitudes Toward Massage
CAM: Complementary and alternative medicine
IRB: Institutional Review Board
REDCap: Research Electronic Data Capture
SAS: Statistical Analysis System_®_
