# Supplementary material for: Massage perceptions and attitudes of undergraduate pre-professional health sciences students: a cross-sectional survey in one U.S. university
Source: BMC Complement Med Ther. 2020 Jul 8;20:213. doi: 10.1186/s12906-020-03002-6 (PMC7346672; doi:10.1186/s12906-020-03002-6)
Supplement: Supplementary file 1 — Additional file 1. [file 12906_2020_3002_MOESM1_ESM.pdf]

Massage Attitude and Perception: Survey Study

1. Have you been asked to take this survey this week?
   - ☐ Yes
   - ☐ No

2. Have you already taken this survey this week?
   - ☐ Yes
   - ☐ No

Massage Attitude and Perception: Survey Study

Thank you for your willingness and interest in completing this survey which is being conducted by xxx the xx School of Health and Human Sciences. Your participation is completely **voluntary**. This survey is designed to gather information about the attitudes and perceptions of Pre-Professional Health Students on Massage Therapy. We realize how precious your time is. That is why we made sure this survey will take approximately 10 minutes. If you have any question, please contact _____ or 317___.
Please fill out the form below as accurately, honestly and completely as possible. There are no right or wrong answers. All of your responses are confidential.

Thank you!

Are you willing to participate in this study?	◯ Yes ◯ No
Are you 18 years old or over?	◯ Yes ◯ No
How old are you?	☐
What is your gender?	◯ Male<br>◯ Female<br>◯ Transgender<br>◯ Other<br>◯ Prefer not to answer
Do you consider yourself as LGBTQQ+?	◯ Yes ◯ No<br>◯ Prefer not to answer
With which racial group(s) do you identify yourself?<br>Please mark all that apply.	☐ White<br>☐ Black or African American<br>☐ Asian<br>☐ American Indian or Alaska Native<br>☐ Native Hawaiian or Other Pacific Islander<br>☐ Other<br>☐ Choose not to answer
Are you of Hispanic or Latino origin?	◯ I am not Hispanic or Latino origin<br>◯ I am of Hispanic or Latino origin<br>◯ Choose not to answer
What is your current class standing?	◯ Freshman<br>◯ Sophomore<br>◯ Junior<br>◯ Senior
Are you a Health Sciences major?	◯ Yes ◯ No
Have you ever had a massage from professional massage therapist?	◯ Yes ◯ No
If you selected "Yes", Please estimate the number of massages, performed by a trained massage therapist, that you have received in your life time?	☐
Please estimate the number of massages, performed by a trained massage therapist, that you have received in the previous twelve-month period?	☐

The following questions are from Moyer et al. 2009 Attitude of Massage Scale.
Please answer each question to the best of your ability.

1. Receiving massage is as good for the mind as it is for the body.

Strongly Disagree | Disagree | Neutral | Agree | Strongly Agree

2. Receiving regular massage would be good for promoting health and well-being.

Strongly Disagree | Disagree | Neutral | Agree | Strongly Agree

3. Massage is a serious form of therapy.

Strongly Disagree | Disagree | Neutral | Agree | Strongly Agree

4. Massage should be covered by health insurance.

Strongly Disagree | Disagree | Neutral | Agree | Strongly Agree

5. I like to be massaged.

Strongly Disagree | Disagree | Neutral | Agree | Strongly Agree

6. Receiving a massage is relaxing.

Strongly Disagree | Disagree | Neutral | Agree | Strongly Agree

7. Receiving a massage would improve my mood.

Strongly Disagree | Disagree | Neutral | Agree | Strongly Agree

8. Receiving a massage would make me nervous.

Strongly Disagree | Disagree | Neutral | Agree | Strongly Agree

9. I like to be touched by other people.

Strongly Disagree | Disagree | Neutral | Agree | Strongly Agree

10. Massage is dirty or inappropriate.

Strongly Disagree | Disagree | Neutral | Agree | Strongly Agree

11. I would prefer that my massage therapist be of the opposite sex.

Strongly Disagree | Disagree | Neutral | Agree | Strongly Agree

12. I am afraid I might become sexually aroused during a massage.

Strongly Disagree | Disagree | Neutral | Agree | Strongly Agree

13. Receiving massage is often sexually arousing.

Strongly Disagree | Disagree | Neutral | Agree | Strongly Agree

14. I would be comfortable receiving massage from a woman.

Strongly Disagree | Disagree | Neutral | Agree | Strongly Agree

15. I would be comfortable receiving massage from a man.

Strongly Disagree | Disagree | Neutral | Agree | Strongly Agree

16. I would prefer that my massage therapist be the same sex as I am.

Strongly Disagree | Disagree | Neutral | Agree | Strongly Agree

We are extremely grateful for contributing your valuable time, and your honest information. If you have any comments on the survey or the project, please leave a comment below.

Many thanks,

ATOM Project Team
