# Supplementary material for: Massage perceptions and attitudes of undergraduate pre-professional health sciences students: a cross-sectional survey in one U.S. university
Source: BMC Complement Med Ther. 2020 Jul 8;20:213. doi: 10.1186/s12906-020-03002-6 (PMC7346672; doi:10.1186/s12906-020-03002-6)
Supplement: Supplementary file 2 — Additional file 2. [file 12906_2020_3002_MOESM2_ESM.docx]

Table 2. ATOM Responses: 9 Item Questions and 7 Supplement Questions.

| **ATOM Items** | Strongly Disagree/  Disagree  n (%) | Neutral  n (%) | Strongly Agree/  Agree  n (%) |
| --- | --- | --- | --- |
| Receiving massage is as good for the mind as it is for the body. | 1 (0.8) | 9 (7.0) | 119 (92.2) |
| Receiving regular massage would be good for promoting health and well-being. | 1 (0.8) | 13 (10.1) | 115 (89.1) |
| Massage is a serious form of therapy. | 6 (4.6) | 21 (16.3) | 102 (79.1) |
| Massage should be covered by health insurance. | 16 (12.4) | 34 (26.4) | 79 (61.2) |
| I like to be massaged. | 6 (4.6) | 21 (16.3) | 102 (79.1) |
| Receiving a massage is relaxing. | 3 (2.3) | 11 (8.5) | 115 (89.2) |
| Receiving a massage would improve my mood. | 4 (3.1) | 15 (11.6) | 110 (85.3) |
| Receiving a massage would make me nervous. * | 93 (72.1) | 21 (16.3) | 15 (11.6) |
| I like to be touched by other people. | 38 (29.5) | 67 (51.9) | 24 (18.6) |
| **Supplementary ATOM Items** |  |  |  |
| I would prefer that my massage therapist be of the opposite sex. | 48 (37.2) | 65 (50.4) | 16 (12.4) |
| I would prefer that my massage therapist be the same sex as I am. | 20 (15.5) | 73 (56.6) | 30 (27.9) |
| I would be comfortable receiving massage from a woman. | 3 (2.3) | 13 (10.1) | 113 (87.6) |
| I would be comfortable receiving massage from a man. | 24 (18.6) | 27 (20.9) | 78 (60.5) |
| Massage is dirty or inappropriate.* | 123 (96.1) | 4 (3.1) | 1 (0.8) |
| I am afraid I might become sexually aroused during a Massage.* | 108 (83.7) | 18 (14.0) | 3 (2.3) |
| Receiving massage is often sexually arousing.* | 95 (73.6) | 31 (24.0) | 3 (2.3) |

**Strongly disagree/Disagree are considered positive feedback.*
