# Supplementary material for: Massage perceptions and attitudes of undergraduate pre-professional health sciences students: a cross-sectional survey in one U.S. university
Source: BMC Complement Med Ther. 2020 Jul 8;20:213. doi: 10.1186/s12906-020-03002-6 (PMC7346672; doi:10.1186/s12906-020-03002-6)
Supplement: Supplementary file 3 — Additional file 3. [file 12906_2020_3002_MOESM3_ESM.docx]

Table 4. Supplemental ATOM Items per Massage Experience Responses: Negative (-), Neutral (=), or Positive (+).

| **Supplementary ATOM Items** | **Massage Experienced**  n (%)  46 (35.7) | **No Massage Experience**  n (%)  83 (64.3) | **P-Value** |
| --- | --- | --- | --- |
| I would prefer that my massage therapist be of the opposite sex.  Disagree/Strongly Disagree  Neutral  Agree/Strongly Agree | 23 (50.0)  17 (37.0)  6 (13.0) | 25 (30.1)  48 (57.8)  10 (12.1) | 0.057 |
| I would prefer that my massage therapist be the same sex as I am.  Disagree/Strongly Disagree  Neutral  Agree/Strongly Agree | 10 (21.7)  19 (41.3)  17 (37.0) | 10 (12.1)  54 (65.1)  19 (22.9) | 0.033 |
| I would be comfortable receiving massage from a woman.  Disagree/Strongly Disagree -  Neutral =  Agree/Strongly Agree + | 2 (4.4)  4 (8.7)  40 (87.0) | 1 (1.2)  9 (10.8)  73 (88.0) | 0.498 |
| I would be comfortable receiving massage from a man.  Disagree/Strongly Disagree -  Neutral =  Agree/Strongly Agree + | 9 (19.6)  10 (21.7)  27 (58.7) | 15 (18.1)  17 (20.5)  51 (61.5) | 0.954 |
| Massage is dirty or inappropriate.  Disagree/Strongly Disagree +  Neutral =  Agree/Strongly Agree - | 43 (95.6)  2 (4.4)  0 (0.0) | 80 (96.4)  2 (2.4)  1 (1.2) | 0.628 |
| I am afraid I might become sexually aroused during a massage.  Disagree/Strongly Disagree +  Neutral =  Agree/Strongly Agree - | 42 (91.3)  3 (6.5)  1 (2.2) | 66 (79.5)  15 (18.1)  2 (2.4) | 0.189 |
| Receiving massage is often sexually arousing.  Disagree/Strongly Disagree +  Neutral =  Agree/Strongly Agree - | 38 (82.6)  7 (15.2)  1 (2.2) | 57 (68.7)  24 (28.9)  2 (2.4) | 0.212 |
