# Supplementary material for: Massage perceptions and attitudes of undergraduate pre-professional health sciences students: a cross-sectional survey in one U.S. university
Source: BMC Complement Med Ther. 2020 Jul 8;20:213. doi: 10.1186/s12906-020-03002-6 (PMC7346672; doi:10.1186/s12906-020-03002-6)
Supplement: Supplementary file 4 — Additional file 4. [file 12906_2020_3002_MOESM4_ESM.docx]

Table 5. Supplemental ATOM Items for Each Gender. Responses: Negative (-), Neutral (=), or Positive (+).

| **Supplementary ATOM Items** | **Male**  n (%)  n=36 (28%) | **Female**  n (%)  n=92 (72%) | **P-Value** |
| --- | --- | --- | --- |
| I would prefer that my massage therapist be of the opposite sex.  Disagree/Strongly Disagree  Neutral  Agree/Strongly Agree | 6 (16.7)  16 (44.4)  14 (38.9) | 41 (44.6)  49 (53.3)  2 (2.2) | 0.0001 |
| I would prefer that my massage therapist be the same sex as I am.  Disagree/Strongly Disagree  Neutral  Agree/Strongly Agree | 15 (41.7)  19 (52.8)  2 (5.6) | 5 (5.4)  53 (57.6)  34 (37.0) | 0.0001 |
| I would be comfortable receiving massage from a woman.  Disagree/Strongly Disagree -  Neutral =  Agree/Strongly Agree + | 1 (2.8)  2 (5.6)  33 (91.7) | 2 (2.2)  11 (12.0)  79 (85.9) | 0.553 |
| I would be comfortable receiving massage from a man.  Disagree/Strongly Disagree -  Neutral =  Agree/Strongly Agree + | 6 (16.7)  5 (13.9)  25 (69.4) | 18 (19.6)  22 (23.9)  52 (56.5) | 0.355 |
| Massage is dirty or inappropriate.  Disagree/Strongly Disagree +  Neutral =  Agree/Strongly Agree - | 35 (97.2)  0 (0.0)  1 (2.8) | 87 (95.6)  4 (4.4)  0 (0.0) | 0.127 |
| I am afraid I might become sexually aroused during a massage.  Disagree/Strongly Disagree +  Neutral =  Agree/Strongly Agree - | 22 (61.1)  13 (36.1)  1 (2.8) | 85 (92.4)  5 (5.4)  2 (2.2) | 0.0001 |
| Receiving massage is often sexually arousing.  Disagree/Strongly Disagree +  Neutral =  Agree/Strongly Agree - | 21 (58.3)  14 (38.9)  1 (2.8) | 73 (79.4)  17 (18.5)  2 (2.2) | 0.0486 |
